# A universal cannabinoid CB1 and CB2 receptor TR-FRET kinetic ligand-binding assay

**DOI:** 10.3389/fphar.2025.1469986

**Published:** 2025-04-09

**Authors:** Leire Borrega-Roman, Bradley L. Hoare, Miroslav Kosar, Roman C. Sarott, Kacper J. Patej, Jara Bouma, Morgan Scott-Dennis, Eline J. Koers, Thais Gazzi, Leonard Mach, Sergio Barrondo, Joan Sallés, Wolfgang Guba, Eric Kusznir, Marc Nazaré, Arne C. Rufer, Uwe Grether, Laura H. Heitman, Erick M. Carreira, David A. Sykes, Dmitry B. Veprintsev

**Affiliations:** ^1^ Division of Physiology, Pharmacology & Neuroscience, School of Life Sciences, University of Nottingham, Nottingham, United Kingdom; ^2^ Centre of Membrane Proteins and Receptors (COMPARE), University of Birmingham and University of Nottingham, Midlands, United Kingdom; ^3^ Department of Pharmacology, Faculty of Pharmacy, University of the Basque Country UPV/EHU, Vitoria-Gasteiz, Spain; ^4^ Bioaraba, Neurofarmacología Celular y Molecular, Vitoria-Gasteiz, Spain; ^5^ Laboratorium für Organische Chemie, Eidgenössische Technische Hochschule Zürich, Zürich, Switzerland; ^6^ Division of Drug Discovery and Safety, Leiden Academic Center for Drug Research, Leiden University and Oncode Institute, Leiden, Netherlands; ^7^ Leibniz-Forschungsinstitut für Molekulare Pharmakologie FMP, Campus BerlinBuch, Berlin, Germany; ^8^ Centro de Investigación Biomédica en Red de Salud Mental (CIBERSAM), Madrid, Spain; ^9^ Roche Pharma Research and Early Development, Roche Innovation Center Basel, F. Hoffmann-La Roche Ltd., Basel, Switzerland

**Keywords:** time-resolved Forster resonance energy transfer- based binding assay, fluorescent ligand, kinetic ligand binding assay, cannabinoid type 1, cannabinoid type 2, cannabinoid receptors, rebinding, ligand depletion

## Abstract

**Introduction:**

The kinetics of ligand binding to G protein-coupled receptors (GPCRs) is an important optimization parameter in drug discovery. Traditional radioligand assays are labor-intensive, preventing their application at the early stages of drug discovery. Fluorescence-based assays offer several advantages, including a possibility to develop a homogeneous format, continuous data collection, and higher throughput. This study sought to develop a fluorescence-based binding assay to investigate ligand-binding kinetics at human cannabinoid type 1 and 2 receptors (CB1R and CB2R).

**Methods:**

We synthesized D77, a novel tracer derived from the non-selective cannabinoid Δ^8^-THC. Using time-resolved Förster resonance energy transfer (TR-FRET), we developed an assay to study ligand-binding kinetics at physiological temperatures. For CB1R, we truncated the first 90 amino acids of its flexible N-terminal domain to reduce the FRET distance between the terbium cryptate (donor) and the fluorescent ligand (acceptor). The full-length CB2R construct was functional without modification due to its shorter N-terminus. The Motulsky–Mahan competition binding model was used to analyze the binding kinetics of the endocannabinoids and several other non-fluorescent ligands.

**Results:**

The D77 tracer showed nanomolar-range affinity for truncated CB1R (CB1R_91-472_) and full-length CB2R (CB2R_1–360_), displaying competitive binding with orthosteric ligands. D77 exhibited rapid dissociation kinetics from both CB1R and CB2R, which were similar to the fastest dissociating reference compounds. This was critical for accurately determining the on- and off-rates of the fastest dissociating compounds. Using D77, we measured the kinetic binding properties of various CB1R and CB2R agonists and antagonists at physiological temperature and sodium ion concentration.

**Discussion:**

The *k*_on_ values for molecules binding to CB1R varied by three orders of magnitude, from the slowest (HU308) to the fastest (rimonabant). A strong correlation between *k*_on_ and affinity was observed for compounds binding to CB1R, indicating that the association rate primarily determines their affinity for CB1R. Unlike CB1R, a stronger correlation was found between the dissociation rate constant *k*_off_ and the affinity for CB2R, suggesting that both *k*_on_ and *k*_off_ dictate the overall affinity for CB2R. Exploring the kinetic parameters of cannabinoid drug candidates could help drug development programs targeting these receptors.

## Introduction

Cannabinoid type 1 and 2 receptors (CB1R and CB2R) are essential signaling elements of the endocannabinoid system. They belong to the G protein-coupled receptor (GPCR) family and show different distribution patterns in the human body. The CB1R is predominantly expressed in the central nervous system (CNS) (Katona et al., 1999), but it is also found in peripheral organs, including adipose tissue, the liver, and the pancreas, where it is involved in the regulation of metabolic functions (Pacher et al., 2006; Han and Kim, 2021). The CB2R is primarily expressed in peripheral tissues related to the immune system (Khurana et al., 2017), such as the spleen and thymus (Munro et al., 1993; Galiègue et al., 2005), but it is also found in the CNS, although expressed at much lower levels compared to CB1R. Efforts to develop drugs targeting CB1R have primarily focused on their roles in neuromodulation and metabolic regulation, particularly in obesity treatment, while CB2R has been explored for its ability to regulate inflammatory processes and immune-related disorders (Aso and Ferrer, 2016; Kaur et al., 2016; Vuic et al., 2022), with further potential to treat or ameliorate certain neurodegenerative disorders (Navarro et al., 2016; An et al., 2020; Bala et al., 2024).

Phytocannabinoids, such as extracts of the plant *Cannabis sativa*, have been used in traditional medicine for millennia (Zuardi, 2006; Zuardi, 2008). Even though the cannabinoid system has been considered a promising intervention point for a plethora of diseases (Di Marzo, 2018; Maccarrone et al., 2023), very few drugs targeting CB1R or CB2R have reached the clinic to date. Currently, dronabinol and nabilone are the only two Food and Drug Administration (FDA)-approved synthetic cannabinoids, which are Δ^9^-THC or close derivatives. These are non-selective compounds used for treating nausea and anorexia, which are the outcomes of cancer chemotherapy treatments. One example of a selective CB1R ligand is the antagonist rimonabant, which was marketed as an effective anti-obesity drug. However, it was subsequently withdrawn from the market due to its adverse neuropsychiatric effects (Moreira and Crippa, 2009).

Difficulties in targeting CB1R and CB2R for therapeutic benefit can be partly explained by their wide distribution in the body and their role as modulators of multiple processes that are primarily driven by other signaling cascades. Designing selective drug candidates with optimal pharmacological properties requires addressing multiple challenges, including receptor-binding kinetics. Although affinity measures are useful, they often fail to fully capture the complexities of drug–receptor interaction. Incorporating kinetic parameters, such as residence time, into the early stages of drug discovery offers valuable insights into drug efficacy, rebinding, and off-target toxicity. This understanding plays a crucial role in enhancing rational drug design (Fuchs et al., 2000; Casarosa et al., 2009; Tresadern et al., 2011; Fleck et al., 2012; Sykes et al., 2012; Guo et al., 2016; Sykes et al., 2017; Sykes et al., 2022). Ligand–receptor kinetic studies also enable better predictions of drug–receptor coverage *in vivo*, which in turn leads to improved therapeutic outcomes (Copeland, 2016; Vauquelin, 2016). Even though our understanding of cannabinoid receptor pharmacology has dramatically increased during the last few decades, the binding kinetics of compounds acting on either of these receptor subtypes under physiologically relevant conditions remain largely unexplored.

Traditionally, drug–receptor binding properties have been investigated by means of radiolabeled ligands, with excellent progress in developing selective ligands for CB2R (Dowling and Charlton, 2009; Sykes et al., 2010; Ramsey et al., 2011; Martella et al., 2017; Soethoudt et al., 2018). However, these methods are very labor-intensive and present many limitations such as lower throughput, the risk associated with the handling of hazardous radioactive material, the increased time and costs incurred by these experimental procedures, and the inability to study rapid binding events (Martella et al., 2017; Xia et al., 2017; Xia et al., 2018; Georgi et al., 2019; Sykes et al., 2019). An important consideration for such filtration-based radioligand binding assays is that the tracer itself must possess a relatively slow-off rate from the receptor of interest so that the effective separation of bound and unbound radioligands is achievable during the washing stage. However, in order to quantify the kinetic parameters of typical CB2R selective ligands, which display fast dissociation kinetics, the tracer itself must also display comparably fast dissociation kinetics (Sykes et al., 2019). Another disadvantage of using radioligands is the higher levels of background signals exhibited by certain probes, even when they possess high affinity for the primary target. The use of fluorescence-based methods and resonance energy transfer, in particular, has helped revolutionize the study of ligand binding to GPCRs, with the main advantage being the lower levels of non-specific signals due to the requirement for proximity between the donor and acceptor in the generation of the specific binding signal (Sykes et al., 2019; Soave et al., 2020). An additional benefit of fluorescence-based time-resolved Förster resonance energy transfer (TR-FRET) assays is that they are homogenous and do not require the separation of bound and unbound ligands. Kinetic binding assays using labeled tracer ligands can also be used to determine the on/off rates of unlabeled ligands at the receptor (Motulsky and Mahan, 1984), provided that the kinetics of the tracer binding are suitable (Sykes et al., 2019). During the course of this work, another group reported the use of TR-FRET binding assays for CB1R and CB2R using a proprietary fluorescent ligand (Raïch et al., 2021; Navarro et al., 2023); however, only equilibrium data were presented, so it is unclear if this probe would be suitable for profiling the kinetic properties of low-affinity rapidly dissociating compounds.

Herein, we report a homogeneous TR-FRET competitive association binding assay using the novel fluoroprobe D77, a nitrobenzoxadiazole (NBD)-labeled tracer, based on the pharmacophore embedded in Δ^8^-THC. The use of this tracer allowed us to characterize CB1R and CB2R ligand kinetic parameters (association rate constant *k*_on_ and dissociation rate constant *k*_off_) at 37°C using the kinetic model of drug–receptor competition binding proposed by Motulsky and Mahan (1984). Using a set of reference compounds for CB1R and CB2R, we present a robust method to perform high-throughput *in vitro* screening of cannabinoid compounds to assess both their affinity and ligand-binding kinetics and explore the basis of selectivity between these two main cannabinoid receptors.

## Materials and methods

### Materials

T-REx™-293 (Invitrogen) cells were obtained from Thermo Fisher Scientific. Culture flasks T75 and T175 cm^2^ were purchased from Thermo Fisher Scientific. DMEM (high glucose) and Dulbecco’s phosphate-buffered saline (DPBS), with no calcium and magnesium (D8537), were purchased from Sigma-Aldrich. Cellstripper™ was purchased from Corning. Hanks’ Balanced Salt solution (H8264), HEPES (4-(2-hydroxyethyl)-1-piperazineethanesulfonic acid), EDTA (ethylenediaminetetraacetic acid), bovine serum albumin (BSA) heat shock fraction, protease-free, fatty acid-free, essentially globulin-free (A7030), poly-D-lysine, tetracycline, and Pluronic F127 were purchased from Sigma-Aldrich. The transfection reagent PEI Linear, MW 25000, transfection-grade (PEI 25K) was obtained from Polysciences (23966-1). The selection reagents blasticidin, geneticin (G418), and zeocin were obtained from Invitrogen. A bicinchoninic acid (BCA)-based protein assay kit (Pierce™ BCA Protein Assay Kit) used to determine the total protein content of membranes was obtained from Thermo Fisher Scientific. 2-AG, AEA, rimonabant, SR 144528, HU-308, and HU-210 were obtained from Tocris Bioscience (United Kingdom). CP 55,940 was obtained from Sigma-Aldrich. All ligands were dissolved in 100% DMSO and stored as aliquots at −20°C until required. Dimethyl sulfoxide (DMSO, 276855) was purchased from Sigma-Aldrich. OptiPlate-384 (white opaque 384-well microplate) was purchased from PerkinElmer (Beaconsfield, United Kingdom).

### Plasmid constructs: cloning and preparation

Plasmid constructs for human CB1R and CB2R have been reported previously (Heydenreich et al., 2017; Hoare et al., 2024). Constructs were in pcDNA4/TO, with a preceding cleaved signal peptide (5HT3A; to target the receptor with an extracellular SNAP tag to the ER for correct membrane incorporation and biogenesis), followed by a Twin-Strep affinity tag, a SNAP tag, and the receptor sequence. To produce the truncated CB1R construct, a PCR-based Gibson assembly method (Heydenreich et al., 2017) was used to remove the relevant nucleotide sequence. All plasmid constructs were verified through the entirety of the coding sequence via Sanger sequencing. Both CB1R and CB2R coding sequences were the canonical human isoform (UniProt ID #P21554-1 for CB1R and #P34972 for CB2R). Plasmid DNA was prepared using standard bacterial expression methods and purified using column-based extraction kits from QIAGEN.

### Cell culture and membrane preparation

T-REx™-293 cells were cultured in T75 and T175 flasks in DMEM (high glucose) supplemented with 10% FBS and blasticidin (10 μg/mL; to maintain the selection pressure for the pcDNA6/TR plasmid, which represses constitutive expression from the CMV promotor of pcDNA4/TO). Cells have been tested for *mycoplasma* using the MycoAlert^®^ PLUS *Mycoplasma* Detection Kit (Lonza). Passage numbers were kept below 20. To generate stable cell lines expressing SNAP-tagged receptors, T-REx™-293 cells were transfected with pcDNA4/TO constructs using PEI and selected with zeocin (20 μg/mL) for 14 days to obtain an antibiotic-resistant cell population with plasmid expression.

T-REx™-293 cells stably expressing either SNAP-CB1R_91-472_ or SNAP-CB2 were cultured in T175 flasks using DMEM supplemented with 10% fetal bovine serum. Tetracycline (1 μg/mL) was added to the culture 48 h prior to labeling to induce receptor expression. When the cells reached 90%–100% confluency, the medium was removed, and the cells were washed using 10 mL of PBS, followed by 10 mL Tag-lite labeling medium (LABMED, Cisbio). Finally, 10 mL of LABMED containing 100 nM of SNAP-Lumi4-Tb was added and incubated for 1 h at 37°C under 5% CO_2_. After removing the labeling solution, PBS was used to wash the cells, which were dissociated using non-enzymatic dissociation buffer. Cells were centrifuged for 5 min (350 × *g*), and pellets were kept at −80°C until membranes were prepared.

For membrane preparation, all steps were conducted at 4°C to avoid tissue degradation. Cells pellets were thawed and resuspended using ice-cold buffer containing 10 mM HEPES and 10 mM EDTA at pH 7.4. The suspension was homogenized using an electrical homogenizer ULTRA-TURRAX (Ika-Werk GmbH, Germany) and subsequently centrifuged at 1,200 *× g* for 5 min. The pellet obtained containing the cell nucleus and other heavy organelles was discarded, and the supernatant was centrifuged for 30 min at 48,000 *× g* at 4°C (Beckman Avanti J-251 ultracentrifuge; Beckman Coulter). The supernatant was discarded, and the pellet was resuspended using the same buffer (10 mM HEPES and 10 mM EDTA, pH 7.4) and centrifuged a second time for 30 min as described above. Finally, the supernatant was discarded, and the pellet was resuspended using ice-cold 10 mM HEPES and 0.1 mM EDTA at pH 7.4. Protein concentration determination was carried out using the Pierce™ BCA Protein Assay Kit (Thermo Fisher) and using BSA as a standard. The final membrane suspension was aliquoted and maintained at −80°C until required for the assays.

### Common procedures used in TR-FRET experiments

Experiments were performed using T-REx™-293 cell membranes expressing SNAP-tagged human CB1R or CB2R (SNAP-CB1R_91–472_ and SNAP-CB2). All the assays were conducted at either 25°C or 37°C in white 384-well OptiPlate plates (PerkinElmer) using the PHERAstar FSX microplate reader (BMG Labtech). The TRF 337/620/520 optic module was used for all assays, except for tracer MKA-136, where the TRF 337/570/490 module was utilized. Each well was excited with four laser flashes, and signal detection was performed with a 100-μs delay, integrating the signal over 700 μs.

Assay buffer comprised HBSS (Hanks’ Balanced Salt Solution) containing 5 mM HEPES, 0.5% BSA, and 0.02% Pluronic F-127 at pH 7.4. BSA (0.5%) and 0.02% Pluronic F-127 were used to minimize the non-specific binding of hydrophobic compounds to plasticware. Membranes were preincubated with 100 μM of GppNHp at 25 or 37°C for 15 min prior to addition to the plate using automatic injectors to ensure early time points could be measured with accuracy. GppNHp is a non-hydrolyzable GTP analog that will bind to G proteins and promote the dissociation of G proteins from the receptor. This way, we promote a single population of receptors (uncoupled from G proteins) avoiding a mixed population (i.e., uncoupled receptors (R) and receptors coupled to GDP bound G proteins, or G proteins alone (RG)). Adding GTP analogs is a common strategy used in GPCR binding studies to ensure a homogeneous single-state receptor population and simplify the analysis.

To control for potential plate position effects, plate layouts were varied between independent experiments. Non-specific (NS) signals at CB1R and CB2R were determined in the presence of saturating concentrations of rimonabant (3 μM) and SR 144528 (1 μM), respectively. D77 stock was prepared at 100x the final concentration, and 0.4 μL was added to the wells. DMSO was held constant at 2% for all assay points, including the saturation and competition binding experiments, as reflected in the text.

### Saturation binding studies

Fluorescent ligand binding to CB1R and CB2R was assessed by homogeneous time-resolved FRET (HTRF) detection, allowing the construction of saturation binding curves. For fluorescent ligand characterization, six concentrations of D77 were chosen ranging from 31.25 to 1,000 nM. Cell membranes containing human CB1R or CB2R (1 μg/well) were added (at t = 0) to the wells in a total volume of 40 μL containing 2% DMSO at 25 or 37°C, with gentle agitation. The non-specific (NS) signal was determined in the presence of saturating concentrations of either rimonabant (3 μM) or SR 144528 (1 μM). The resulting data were fitted to the one-site model equation (Equation 1) to derive a single best-fit estimate for *K*_d_, as described under *Data analysis*. The receptor concentration in our assay was calculated to be 150–300 pM based on a terbium standard curve (see Supplementary Figure S1). This ensures that ligand depletion to the receptor is minimal as significant depletion will occur when the receptor concentration approaches or exceeds the free ligand concentration and is near its *K*_d_. Ligand depletion is considered significant when more than 10% of the ligand is bound (Motulsky and Neubig, 2002), which, in this case would require nmol-range high-affinity binding sites per µg of protein (assuming negligible non-specific binding in the assay).

One inherent limitation of a 384-well fluorescent ligand binding assay format is the inability to accurately measure ligand depletion. Although the affinity of the tracer used in this study is relatively low, it is crucial to ensure that ligand depletion does not significantly impact the assay, particularly given the small reaction volumes used. The addition of BSA and Pluronic F-127 to the assay buffer minimizes ligand adsorption to the plate surface. The fluorescence intensity of different fluorescent ligand concentrations can be measured to assess this practically, and should be a linear relationship. To further assess potential issues, one approach is to verify that the Hill coefficient of the ligand-binding equilibrium saturation binding curve, when plotted in logarithmic units and fitted to a sigmoidal model, is close to 1 (Carter et al., 2007). Another strategy involves centrifuging the reaction mixture to separate membrane-bound components and confirming that residual fluorescence remains unchanged, helping identify substantial non-specific membrane binding (Hulme, 1999). These considerations are recommended when working with higher-affinity tracers to ensure the validity of binding models that assume equivalence between total and free ligand concentrations.

### Determination of affinity constants (*K*_i_)

To obtain affinity estimates of the unlabeled ligand, D77 competition experiments were performed at equilibrium. D77 was used at a concentration of 600 nM and 900 nM in binding assays for CB1R or CB2R, respectively. D77 was incubated in the presence of the indicated concentration of the unlabeled ligand and CB1 and CB2R cell membranes (1 μg/well) in a total volume of 40 μL containing 2% DMSO at 25 or 37°C, with gentle agitation. The non-specific (NS) signal was determined in the presence of saturating concentrations of either rimonabant (3 μM) or SR 144528 (1 μM). Steady-state competition curves were obtained following a 15-min incubation period, and data were fitted using GraphPad Prism 9.2 to the one site competition binding model (Equation 2) to calculate the IC_50_ values, which were converted to *K*_i_ values by applying the Cheng–Prusoff correction as described under *Data analysis*.

### Determination of the association rate (*k*_on_) and dissociation rate (*k*_off_) of fluorescent ligands

Fluorescent ligand binding to CB1R and CB2R was assessed via HTRF detection, allowing the construction of association binding curves. For fluorescent ligand characterization, six increasing concentrations of fluorescent ligands were prepared, and cell membranes containing human CB1R or CB2R (1 μg/well) were added (at t = 0) in a total volume of 40 μL containing 2% DMSO at 25 or 37°C, with gentle agitation. Non-specific (NS) signals were determined in the presence of saturating concentrations of either rimonabant (3 μM) or SR 144528 (1 μM). As the wells are read sequentially, we restricted the readout of kinetic data of two ligands per experimental run to reduce the minimum reading cycle time. The wells were read every 8 s during a 15-min period, and the resulting data were globally fitted to the association kinetic model (Equation 3) to derive a single best-fit estimate for *K*_d_, *k*_on_, and *k*_off_ as described under *Data analysis*.

### Competition binding kinetics

Competitive kinetic association experiments were carried out using the D77 fluorescent ligand as a tracer. Cell membranes containing the human CB1R or CB2R (1 μg/well) were added (at t = 0) to wells containing D77 and the unlabeled compound to white 384-well OptiPlates in a total volume of 40 μL containing 2% DMSO at 25 or 37°C, with gentle agitation. The concentrations of the tracer used in the experiments were 600 nM and 900 nM for CB1R and CB2R, respectively, which avoid ligand depletion in the volume mentioned above. Concentrations of D77 were selected just above its *K*_d_ for CB1R, taking into account the kinetic properties of the tracer that dictate the time to equilibrium. In this case, using 600 nM allows for a fast equilibrium for the application of the Motulsky and Mahan model. In the case of CB2R, we decided to choose a higher concentration (∼2.5x *K*_d_) in order to compensate for its slower *k*_on_ and lower specific binding HTRF signal. By using 900 nM, we could improve our assay window and accelerate *k*_obs_, which is a function of the ligand concentration used (*k*_obs_ = *k*_on*_[L]+ *k*_off_).

The degree of D77 bound to the receptor was assessed every 8 s over a 15-min period. Non-specific (NS) signals were determined in the presence of saturating concentrations of either rimonabant (3 μM) or SR 144528 (1 μM) and was subtracted from each timepoint. Rimonabant and SR 144528 concentrations used to define NSB were chosen based on the measured p*K*_d_ values of 4.6 nM and 2.82 nM, respectively. These concentrations are >300x the respective *K*_d_ of each ligand and lead to >99.7% of receptor occupation (% Occupied=([L]/([L]+*K*_d_) × 100)). Therefore, the concentrations were chosen to efficiently block fluorescent ligand binding while not causing more general physical changes to the membrane that might alter specific binding.

The association of the D77 was monitored using TR-FRET in competition with 8 different concentrations of each cannabinoid ligand. The resulting data were globally fitted in GraphPad Prism 9.2 to the “kinetics of competitive binding” model (Equation 4) to derive a single best-fit estimate of *K*_d_, *k*_on_, and *k*_off_ for different cannabinoid compounds as described under *Data analysis*.

### Synthesis of fluorescent probes

The structures (see Supplementary Figure S6) and details of synthetic procedures, as well as compound characterizations, can be found in Supplementary Information.

### Material availability

The D77 compound, receptor constructs, and associated cell lines are not commercially available. However, detailed methods for their generation and use are provided within the manuscript.

### Data analysis

Data are expressed in the text and tables as mean ± SEM for the indicated number of experiments. All experiments were analyzed by non-linear regression using Prism 9.2 (GraphPad Software, San Diego, United States).

### Saturation binding

D77 total and non-specific (NS) signal data were analyzed via non-linear regression according to one-site binding equations, and individual estimates for maximal specific binding (*B*_max_) and ligand dissociation constant (*K*_d_) were calculated. The following one-site model equation was used, where [A] is the concentration of the ligand:

$$\begin{array}{c} Specific=\frac{Bmax\left[A\right]}{K_{d}+\left[A\right]}\\ NS=slope\left[A\right]+background \end{array}.$$ (1)

### Competition binding

Steady-state competition displacement binding data were fitted to sigmoidal (variable slope) curves using a “four parameter logistic equation:”

$$Y=Bottom+\frac{\left(Top-Bottom\right)}{1+10^{\left(logIC50-X\right)*Hillslope}}$$ (2)

where Bottom and Top represent the lower and upper plateaus of the specific binding signal, respectively. LogIC_50_ is the logarithm of the competitor concentration that displaces 50% of the bound fluorescent tracer, and the Hill slope is the unitless slope factor. IC_50_ values obtained from the competition curves were converted to *K*_i_ values using the method of Cheng and Prusoff (1973).

### Association binding

D77 association data were fitted to a global fitting model using GraphPad Prism 9.2 to simultaneously calculate *k*_on_ and *k*_off_ using the following equation, where *k*_obs_ equals the observed rate of association and L is the concentration of D77:

$$k_{obs}=\left[L\right]\cdot k_{on}+k_{off}$$ (3)

### Competition kinetic binding

The association rate *k*_on_ and the dissociation rate *k*_off_ calculated for different cannabinoid compounds were obtained by global fitting of competition binding data using the model “kinetics of competitive binding” in GraphPad Prism 9.2:

$$\begin{array}{c} K_{A}=k_{1}\left[L\right]+k_{2}\\ K_{B}=k_{3}\left[I\right]+k_{4}\\ S=\sqrt{\left({\left(K_{A}-K_{A}\right)}^{2}+4\cdot {\;k}_{1}\cdot k_{3}\cdot L\cdot I\cdot 10^{-18}\;\right)}\\ K_{F}=0.5*\left(K_{A}+K_{B}+S\right)\\ K_{S}=0.5*\left(K_{A}+K_{B}-S\right)\\ Q=\frac{{\;B}_{\max}*\;K_{1}*\;L*10^{-9}\;}{K_{F}-K_{S}}\\ Y=Q\cdot \left(\frac{k_{4}\;\cdot \left(K_{F}-K_{S}\right)}{K_{F}\;\cdot \;K_{S}}+\frac{k_{4}-K_{F}}{\;K_{F}}\;\exp^{\left(-K_{F}\;\cdot X\right)}-\frac{k_{4}\;K_{S}}{\;K_{S}}\;\exp^{\left(-K_{S}\;\cdot X\right)}\right) \end{array}$$ (4)

where X is the time (min), Y is the specific binding (HTRF units 520 nm/620 nm*10,000), *k*_1_ is the *k*_on_ of the tracer D77, *k*_2_ is the *k*_off_ of the tracer D77, *L* is the concentration of D77 used (nM), and *I* is the concentration of the unlabeled agonist (nM). Fixing the abovementioned parameters allowed the following to be simultaneously calculated: B_max_ is the total binding (HTRF units 520 nm/620 nm*10,000), *k*_3_ is the association rate of the unlabeled ligand (M^−1^ min^−1^) or *k*_on_, and *k*_4_ is the dissociation rate of the unlabeled ligand (min^–1^) or *k*_off_.

### Linear correlations

The correlation between datasets was determined by calculating the Pearson correlation coefficient (presented as the r^2^ coefficient of determination, which shows percentage variation in y, which is explained by all the x variables together) in GraphPad Prism 9.2.

## Results

### Developing a TR-FRET binding assay for SNAP-CB1R required truncation of the N-terminus

We have previously developed a TR-FRET-based binding assay for human CB2R using a genetically encoded SNAP-tag at the N-terminus of the full-length CB2R along with complementary fluorescent probes (Sarott et al., 2020; Gazzi et al., 2022; Kosar et al., 2023). Although these compounds showed good selectivity for CB2R, as measured in radioligand binding assays, they also bound CB1R, albeit with reduced affinity. However, we found that we could not obtain a TR-FRET signal for CB1R.

**FIGURE 1:**
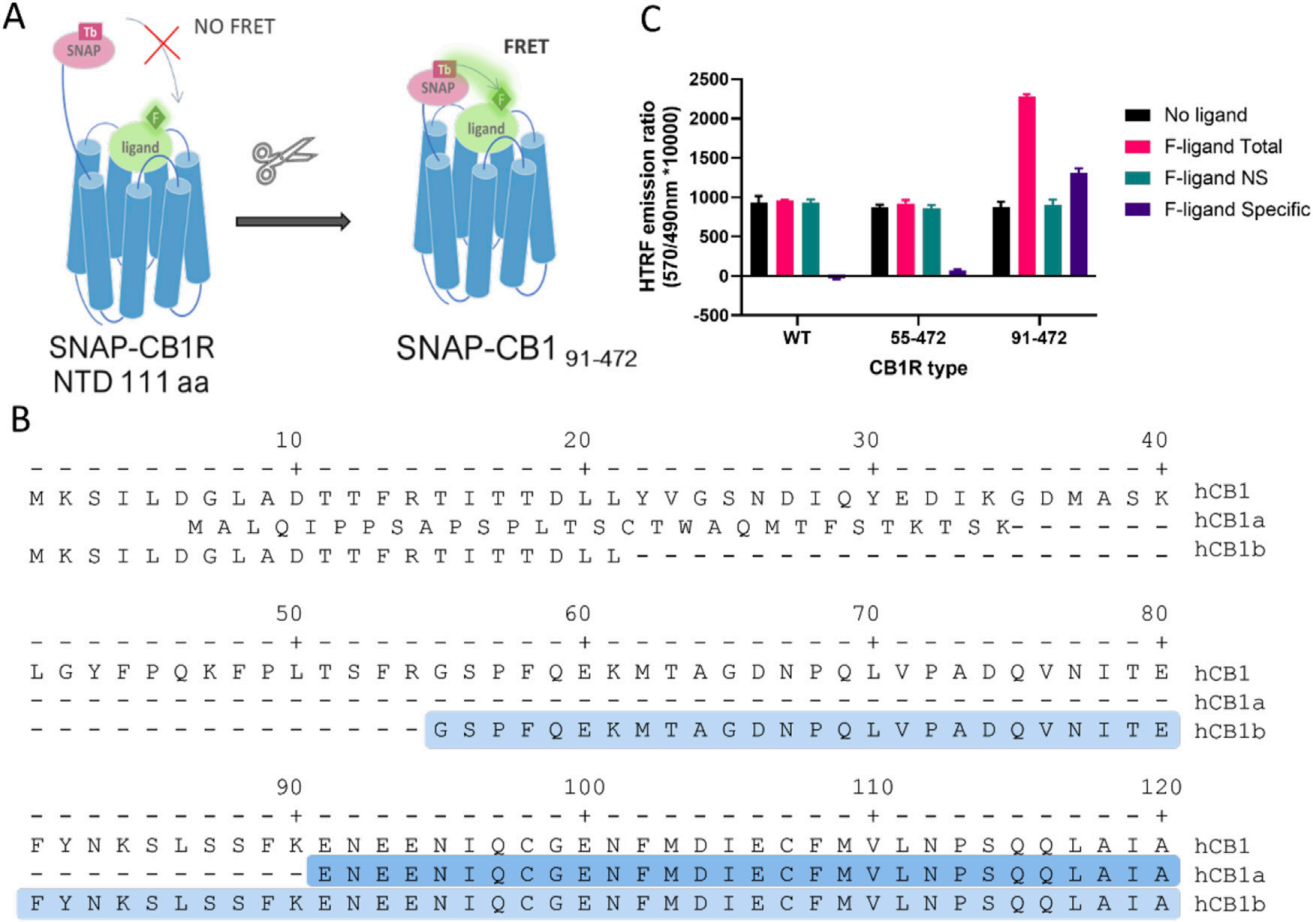
Truncation of human cannabinoid receptor type 1 (CB1R) allows for specific TR-FRET binding signals from fluorescent tracers. **(A)** Diagram showing how the long N-terminal domain (NTD) of CB1R (111 amino acids) may preclude FRET between the terbium cryptate donor and fluorescent tracer and rationale for truncating to shorten the distance. **(B)** Amino acid sequence of the N-terminal of human CB1R and its splicing variants hCB1a and hCB1b. The highlighted amino acids represent the N-terminal sequence of the truncated CB1R versions used (91–472 in dark blue and 55–472 in light blue), modified from Ryberg et al. (2005). **(C)** Total, non-specific (NS), and specific TR-FRET binding signals at full-length and truncated CB1R obtained using NBD-691 (1 µM).

We hypothesized that the 111-amino acid residue long N-terminus of CB1R, as opposed to the 33-residue short N-terminus of CB2R, places the SNAP-Lumi4 donor too far from the fluorescent ligand bound in the orthosteric binding site (Figure 1A) for efficient FRET, at a distance of approximately 60–70 Å (or 6–7 nm). We, therefore, truncated the CB1 N-terminus to place the SNAP tag closer to the binding pocket. The truncation site was rationally chosen based on the knowledge of reported CB1R splicing variants, which have modified N-terminal domains (NTD) and retain functionality (Xiao et al., 2008). Two truncated CB1R variants were produced and named based on the residues remaining after truncation—CB1R_55–472_ and CB1R_91–472_ (Figure 1B), both containing an N-terminal SNAP tag for terbium cryptate labeling. In TR-FRET experiments, which tested for specific binding of the non-selective cannabinoid tracer NBD-691 (1 µM), only the most truncated receptor, CB1R_91–472_, displayed a specific TR-FRET signal that is indicative of ligand binding (Figure 1C). More details on NBD-691 can be found in the Supplementary File.

### Truncated receptor retains cell-surface localization and functionality

Earlier reports suggested that the N-terminal domain of the CB1R may be playing a role in receptor trafficking and stability (Hebert-Chatelain et al., 2016; Fletcher-Jones et al., 2020). In our case, we found that truncated CB1R was still detectably expressed at the cell surface when transiently expressed in HEK293T cells (see Supplementary Methods). Most likely, the signal peptide derived from the 5HT_3A_ receptor and the SNAP tag (a large extracellular folded protein domain) ensured trafficking of the engineered receptor to the cell surface (see Supplementary Figure S2).

Few studies have specifically investigated the role of the NTD of CB1R in influencing its pharmacological properties. Earlier findings suggested that the N-terminus might play a role in binding and signaling for certain endogenous ligands (Ryberg et al., 2005); however, these results have been contradicted by a more recent study reporting no functional differences between splice variants with varying N-terminal lengths (Xiao et al., 2008). To clarify this question, a comprehensive pharmacological comparison of the truncated and full-length CB1R was conducted to assess the impact of NTD truncation on receptor function (i.e., the removal of the first 90 amino acids, CB1R_91–472_). Radioligand binding studies with the tritiated synthetic cannabinoid CP 55,940 (see Supplementary Methods) strongly suggest that CB1R truncation had no substantial effects on synthetic cannabinoid binding (see Supplementary Figure S3; Supplementary Table S1). In addition, the functional effects of the synthetic cannabinoid HU-210 and the endocannabinoids 2-AG and AEA were indistinguishable between truncated and native receptors when tested in intact cells expressing a Gi-CASE activation biosensor (Schihada et al., 2021; Scott-Dennis et al., 2023). This suggests that neither the efficacy (intrinsic activity) nor the potency of the synthetic cannabinoids nor endocannabinoids was affected by truncation of the CB1R (see Supplementary Figure S4; Supplementary Table S2).

### Previously developed fluorescent tracers are too slow or too fast for measurements of ligand-binding kinetics

**FIGURE 2:**
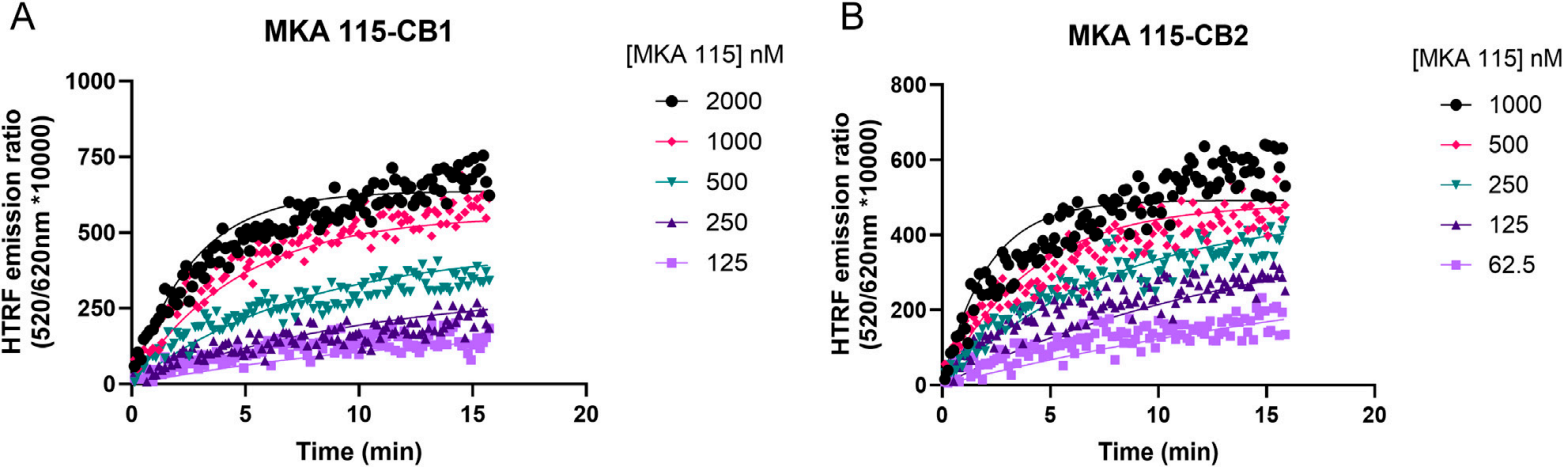
Example kinetic profile of a non-ideal nonselective cannabinoid probe. Kinetic association binding curves of the fluorescent cannabinoid ligand MKA 115 binding to **(A)** CB1R- and **(B)** CB2R-expressing membranes conducted at 25°C. Graphs are representatives of three independent experiments and show specific binding expressed as HTRF emission ratios.

The binding kinetic profile (the association rate *k*_on_ and the dissociation rate *k*_off_) of the tracer molecule will significantly influence the performance of the Motulsky and Mahan model (Georgi et al., 2019; Sykes et al., 2019). A previously developed tracer based on HU-308, MKA-115, exhibited a slow rate of association to both CB1R and CB2R. Since fast association is desired to observe competition with the cold compounds at the very earliest time points, MKA-115 was not suitable for use (see Figure 2). In addition, MKA-115 failed to reach equilibrium in the 15-min data collection period, meaning that reliable kinetic parameters could not be obtained for this tracer. NBD-691, on the other hand, was binding too fast to CB1R, for its binding kinetics to be measurable by the plate reader. In total, 10 tracers were designed, synthesized, and tested (see Supplementary Figures S5, S6; Supplementary Table S3). The design of our tracers was inspired by a reverse-design approach (Guberman et al., 2022), where known CBR ligands with varied affinity and selectivity toward CB1R and CB2R were used. The ligands were functionalized with alkyl and PEG linkers and different fluorophores to achieve optimal affinity and signal-to-noise ratio with the terbium FRET donor. This process led us to a promising lead—a Δ^8^-THC core with an NBD fluorophore. Precise structural modifications, such as choosing the lipophilic n-heptyl tail of D77 over the n-pentyl tail of MKA-136, enabled candidate D77 to exhibit an ideal profile for our intended application as a universal CBR tracer. Most tracers exhibit significant limitations, such as signal instability caused by photobleaching or an excessively rapid association phase due to very fast *k*_off_ values, which result in the ligand binding reaching equilibrium almost instantaneously. In contrast, D77 demonstrated a moderately rapid *k*_off_, enabling the observation of the association phase before equilibrium. This is critical as the Motulsky and Mahan model depends on the competitive interaction of cold (non-fluorescent) ligands during the tracer’s association phase.

### Synthesis of tracer D77 (NBD-Δ^8^-THC)

Our goal of designing a universal fluorescent probe to determine the pharmacology of CBR specific ligands necessitated the identification of a pharmacophore with balanced affinity for both CB1R and CB2R. In addition, the determination of kinetic parameters of lower-affinity CBR specific ligands, such as the endocannabinoids, necessitates the use of a rapidly dissociating tracer. These criteria are in contrast to our previously reported highly CB2R-selective fluorescent probes (Sarott et al., 2020; Kosar et al., 2024). Δ^8^-THC fulfills this criterion and exhibits good affinity for both CB1R and CB2R, providing an ideal starting point for our studies (Soethoudt et al., 2017). Phenol modification is well-known to sharply reduce the affinity of THC derivatives for CB1R and was thus unsuitable for linker attachment for our purposes (Compton et al., 2002). In contrast, chemical probes based on the THC scaffold, as well as the available SAR data, indicate THC carbon 11 (C(11)) is a suitable locus for attachment of linkers extending into the extracellular matrix, thereby providing a sound basis for the incorporation of fluorophores (Thakur et al., 2005; Papahatjis et al., 2006; Muppidi et al., 2018). We have previously shown the requirement for minimally six carbon atoms to reach the extracellular space, as well as the pharmacological superiority of C(11) amide-over-ester linkages in cannabinoid–fluorophore conjugates, both considerations being accounted for in our design of the FRET tracer D77 (Westphal et al., 2020). Additionally, D77 harbors a C(3) n-heptyl chain, which has recently been shown to enhance the affinity for both CB1 and CB2R when compared to the n-pentyl side chain in THC (Citti et al., 2019). As a fluorescent moiety, we have chosen nitrobenzoxadiazole (NBD). Although NBD has a lower extinction coefficient (ca 20,000 cm^−1^M^−1^) and quantum yield (ca 0.4) (Uchiyama et al., 2003) than dyes like fluorescein (65,000 cm^−1^M^−1^, 0.98), it has been proven to be a good acceptor for the terbium cryptate Lumi4 donor in our hands (Kosar et al., 2023).

**FIGURE 3:**
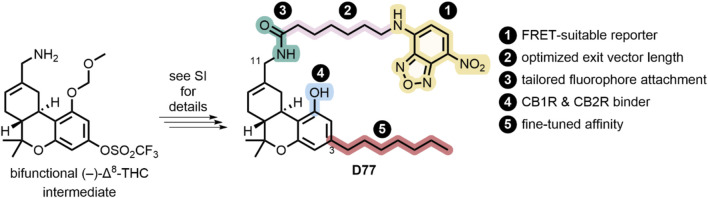
Synthesis and design of D77, a non-selective fluorescent ligand targeting CB1R and CB2R.

The modular synthetic strategy and the structure of our universal cannabinoid receptor probe D77 are shown in Figure 3 with the synthetic methodology described in Supplementary Scheme S1. Once D77 was identified as a suitable tracer, a novel convergent synthetic approach was designed and optimized, starting from commercially available spherophorol, to access D77 in three steps with 34% overall yield (Supplementary Scheme S2). This dramatically simplified the synthesis of D77 (3 vs. 14 steps) and increased the yield of the synthesis from <1% to 34%. The new streamlined and highly efficient synthesis pathway enables a steady supply of the D77 tracer for screening purposes. D77 was characterized, and its identity was confirmed via ^1^H NMR, ^13^C NMR, IR, and MS. The purity of D77 was assessed via ^1^H NMR and UV–Vis (254 nm and 480 nm) (see Supplementary Material). Purity of D77 was >95% across all the analytical tests conducted.

### Characterization of the saturation binding of D77 to CB1R and CB2R

**FIGURE 4:**
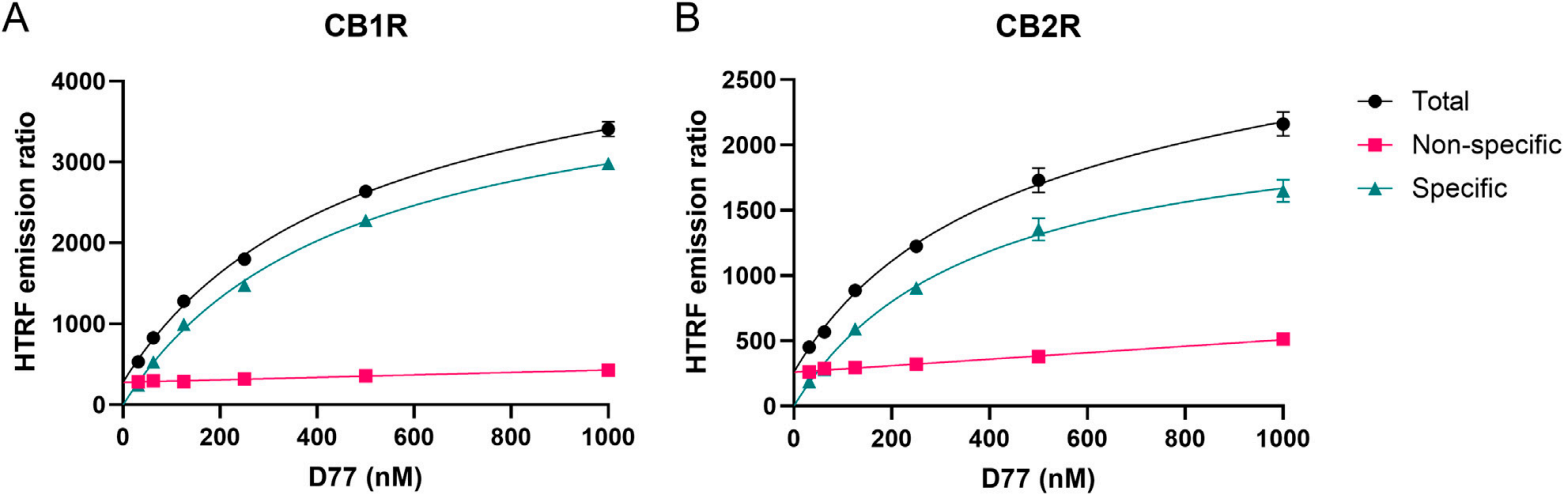
Saturation binding curves at 5 min for D77 ligand binding to CB1R and CB2R at 37°C. Total, non-specific, and specific signal saturation curves are shown for D77 ligand binding to CB1R **(A)** and CB2R **(B)** expressed as the HTRF emission ratio. Non-specific binding was defined in the presence of 3 μM of rimonabant and 1 μM of SR144,528 for CB1R and CB2R, respectively. Graphs are representatives of three independent experiments and represent mean ± SEM of six technical replicates.

Saturation binding experiments with increasing concentrations of D77 were carried out in order to determine the affinity of D77 for the two cannabinoid receptor subtypes. Measurements at steady-state, obtained 5 min after ligand addition, were used as an endpoint to obtain an equilibrium affinity measurement, or *K*_d_ value at each receptor (see Figure 4). Notably, the level of non-specific binding signals was low at both receptors, representing less than 25% of the total binding signal in both cases. Consequently, D77 achieves a relatively high level of specific binding, making it a useful tracer for competition studies and a practical alternative to the high-affinity, agonist radioligands used in the past, which are routinely employed in the absence of guanine nucleotides to preserve more specific binding. Equilibrium binding affinity values for D77 binding to the two CBR subtypes are reported in Table 1. To assess the potential for ligand depletion, we have replotted the saturation curves on a log scale and refitted the data using a four-parameter logistic equation to determine the Hill coefficient (see Supplementary Figure S7). The resulting Hill slopes were close to unity, indicating that no ligand depletion occurred under the assay conditions employed.

**TABLE 1:** Affinity values and kinetic parameters *k*_on_ and *k*_off_ of the D77 fluorescent tracer at 37°C. Values are calculated from kinetic association experiments and saturation experiments performed at equilibrium. Data shown are mean ± SEM of four experiments conducted independently.

	p*K*_d_	Equilibrium p*K*_d_
CB1R	6.36 ± 0.02	6.37 ± 0.02
CB2R	6.48 ± 0.03	6.43 ± 0.04

	*k*_on_ (M^−1^ min ^−1^)	*k*_off_ (min ^−1^)
CB1R	(4.31 ± 0.19) × 10^6^	1.87 ± 0.05
CB2R	(3.46 ± 0.22) × 10^6^	1.13 ± 0.06

### Equilibrium competition experiments using D77

**FIGURE 5:**
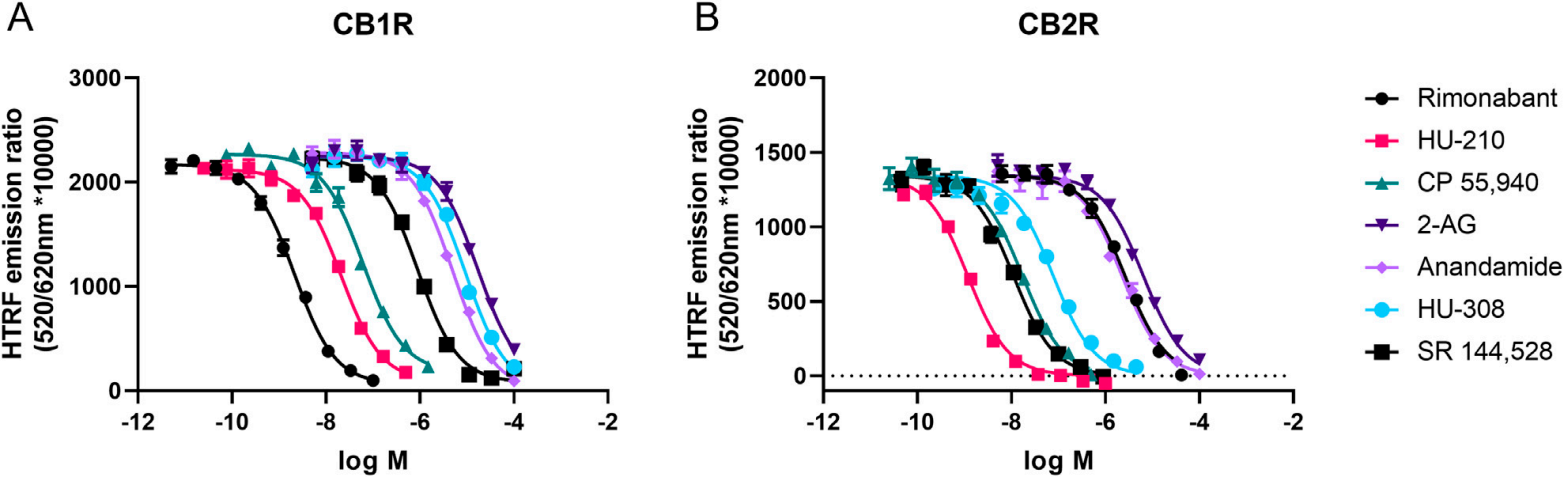
Steady-state competition binding curves of cannabinoid ligands competing with the tracer D77 for CB1R and CB2R. Experiments were conducted using a fixed concentration of the tracer molecule D77 and increasing concentrations of unlabeled ligands using membranes expressing **(A)** CB1R and **(B)** CB2R. Competition binding curves for the cannabinoid compounds are shown for both receptors at 37°C and following a 15-min incubation period. Graphs are representatives of three independent experiments and represent mean ± SEM of six technical replicates.

Equilibrium competition experiments allowed us to calculate the equilibrium dissociation constant (or p*K*_i_ values) of our test set of cannabinoid compounds under equilibrium conditions. To achieve this aim, binding of D77 to CB1R and CB2R was monitored using TR-FRET in the presence of increasing concentrations of cannabinoid ligands, and IC_50_ parameters were obtained from the derived curves, as shown in Figure 5. The Cheng–Prusoff conversion equation was used to calculate *K*_i_ values from the IC_50_ values derived from these inhibitory curves, and these values expressed as negative logarithms can be found in Table 2. Generally, the p*K*_i_ values obtained using D77 were in good agreement with the literature (Govaerts et al., 2004; Khajehali et al., 2015; Martella et al., 2017; Soethoudt et al., 2017). The values obtained in this study for endogenous ligands 2-AG or AEA were somewhat lower compared to those reported by Soethoudt et al. (2017), but were comparable with the aggregated results collected by multiple laboratories (reviewed in Carruthers and Grimsey, 2023) for CB2R. A possible source of the variation in the reported values is the chemical instability of the 2-AG or AEA, which are degraded by enzymes naturally present in the cell membranes, as well as variations in the assays, where the high-affinity state caused by the G protein binding was retained or specifically dissociated by the addition of GTP analogs.

**TABLE 2:** Equilibrium dissociation constant of the cannabinoid ligands tested expressed as p*K*_i_ at 37°C. The data shown are mean ± SEM from four experiments conducted independently in singlet.

	p*K*_i_	
	CB1R	CB2R
Rimonabant	8.98 ± 0.05	6.00 ± 0.02
HU-210	7.99 ± 0.01	9.56 ± 0.09
CP 55,940	7.51 ± 0.03	8.32 ± 0.04
2-AG	5.15 ± 0.26	5.81 ± 0.03
Anandamide	5.59 ± 0.04	6.11 ± 0.02
HU-308	5.41 ± 0.02	7.57 ± 0.03
SR 144528	6.43 ± 0.01	8.55 ± 0.05

### Kinetics of the association of D77 binding CB1R and CB2R

**FIGURE 6:**
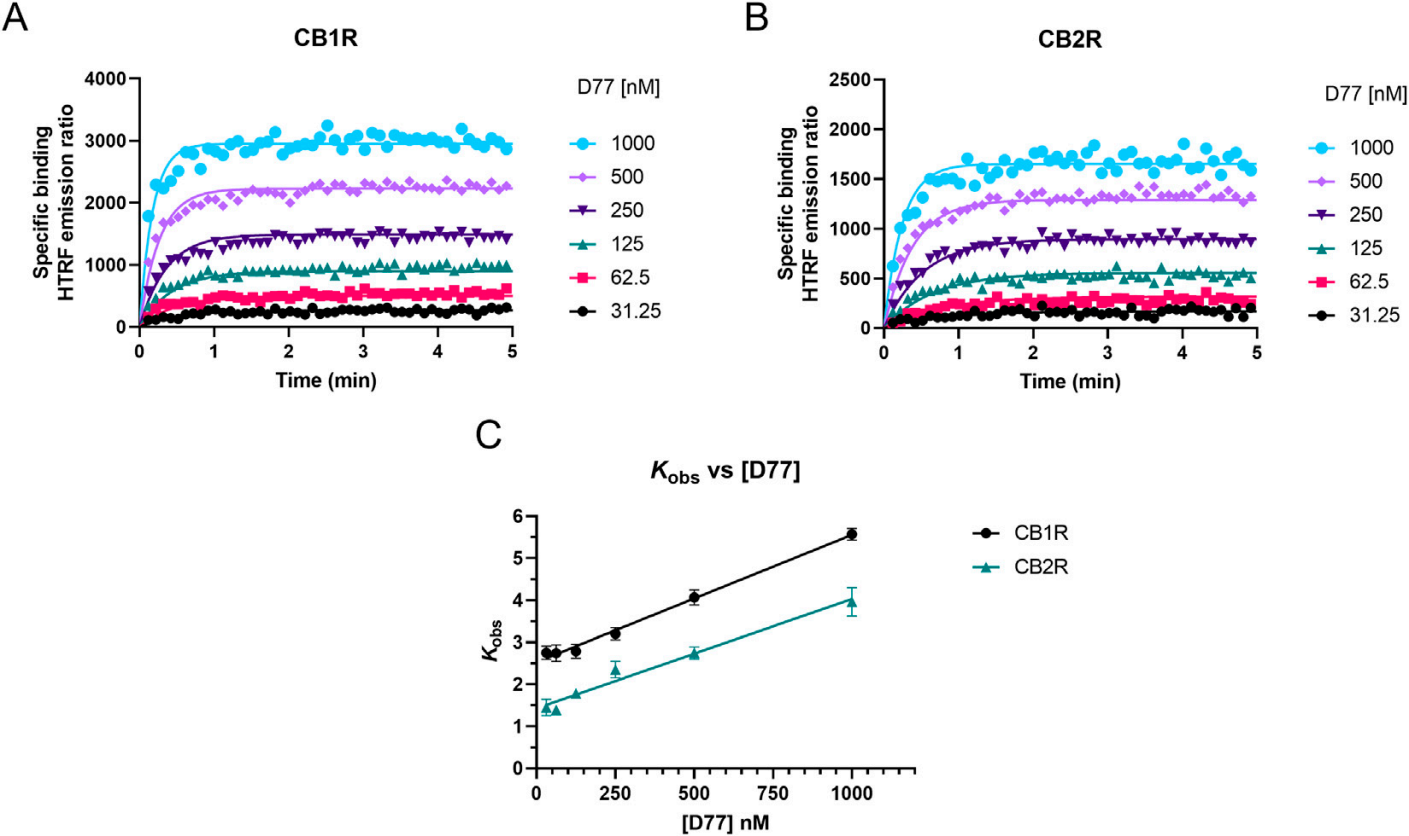
Determination of D77 kinetic binding parameters at 37°C. Association binding curves obtained from six concentrations of the fluorescent ligand D77 are shown for **(A)** CB1R and **(B)** CB2R. Kinetic experiments derive a *K*_d_ value for the D77 ligand of 437 ± 22 nM for CB1R and 370 ± 16 nM for CB2R. The observed association rate constant (*k*_obs_) obtained for each concentration of fluorescent tracer D77 fitted to a linear regression model **(C)**. Binding followed a simple law of mass action model, and *k*_obs_ increasing in a linear manner with fluorescent ligand concentration. Data are presented as representative graphs **(A, B)** or mean ± SEM **(C)** from four experiments conducted independently.

We measured the real-time association of the fluorescent tracer D77 to both cannabinoid receptor subtypes at 37°C over a period of 5 min using TR-FRET. D77 showed a rapid association profile to both CB1R and CB2R, reaching equilibrium within the first 2 min (see Figure 6).

As shown in Figures 6A, B, D77 shows optimal binding characteristics at CB1R and CB2R, making it an ideal tracer for the Motulsky and Mahan approach, which is used to determine the kinetics of unlabeled compounds binding these receptors. D77 shows a relatively fast association profile (*k*_obs_ = (*k*_on_ × L) + *k*_off_), yet it still allows the accurate determination of the associating phase prior to equilibrium, which is necessary for the application of the competitive association binding model (see Figures 6A, B). The association rate constants measured for D77 were relatively slow at both receptors (*k*_on_-CB1R= (4.3 ± 0.2) × 10^6^ M^−1^ min^−1^; *k*_on_-CB2R = (3.5 ± 0.2) × 10^6^ M^−1^ min^−1^). However, D77 exhibits fast dissociation rate constants at both cannabinoid receptors, with *k*_off_ values of 1.87 ± 0.05 min^−1^ and 1.13 ± 0.06 min^−1^ being obtained at CB1R and CB2R, respectively, meaning equilibrium between the receptor and tracer is achieved rapidly at both receptor subtypes.

Moreover, the specific binding signal of D77 remains constant over time, and we do not observe any decay in the signal due to signal bleaching within the time frame of data acquisition. The affinity values obtained from saturation equilibrium experiments were in good agreement with the kinetic *K*_d_ values determined using the kinetic approach (where, *K*_d_ = *k*_off_/*k*_on_) (Table 1), demonstrating the reliability of the tracer kinetic model fitting.

As shown in Figure 6C, the observed associated rate (*k*_obs_) increases linearly with fluorescent ligand concentrations, demonstrating the expected relationship for a reversible bimolecular binding interaction (Motulsky and Christopoulos, 2004). The extrapolation of the fitted line to Y = 0 yields to an estimation of *k*_off_ values of 2.53 min^−1^ and 1.43 min^−1^ at CB1R and CB2R, respectively, and the slope indicates a *k*_on_ value at a CB1R of 3.03 × 10^6^ M^−1^ min^−1^ and at a CB2R of 2.60 × 10^6^ M^−1^ min^−1^. These values are in good agreement with those obtained from global fitting of the D77 association binding curves (see Table 1).

### Competition association binding

**FIGURE 7:**
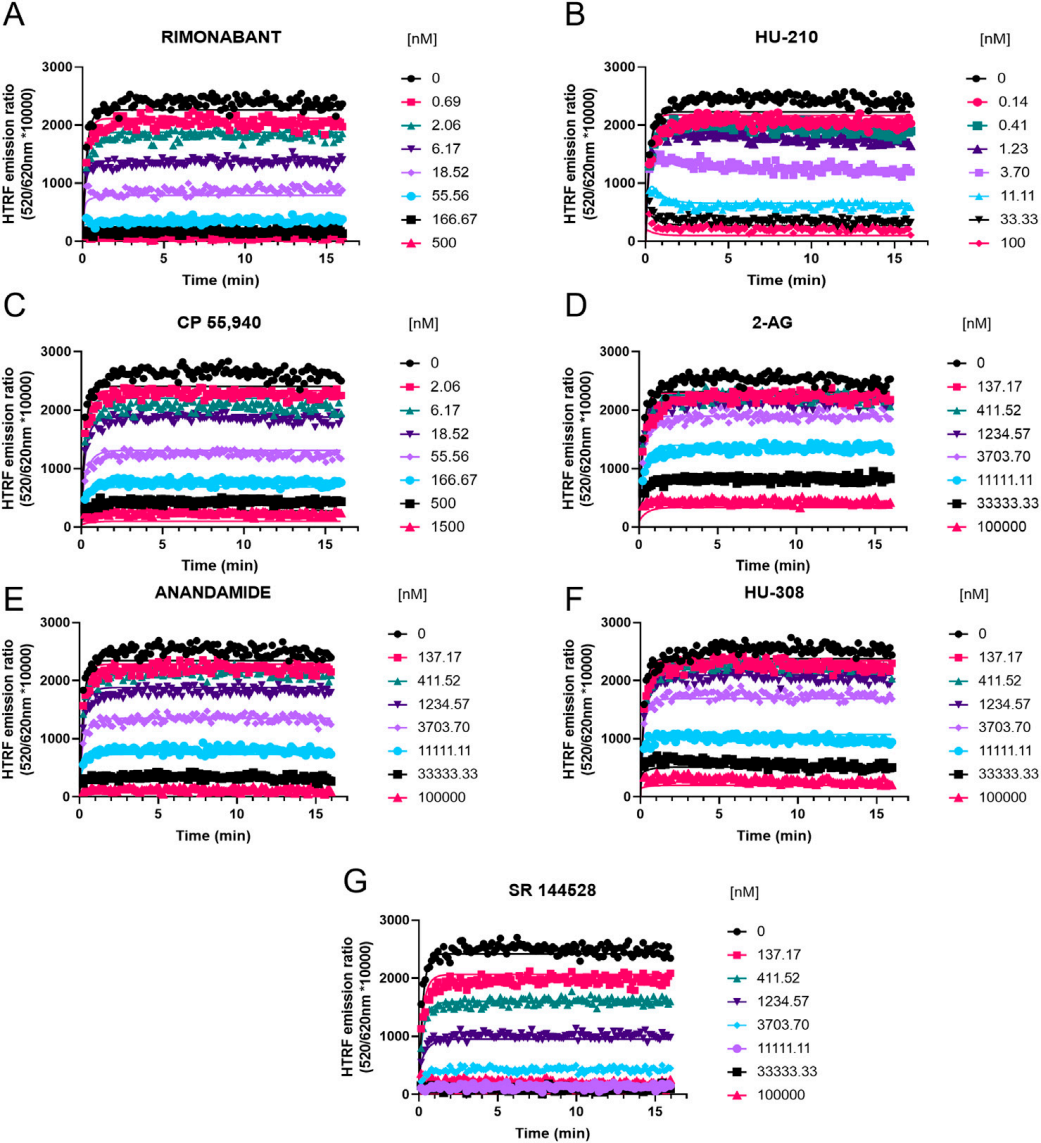
Competition kinetic binding curves of cannabinoid ligands at 37°C competing with D77 for CB1R. Experiments were conducted using a fixed concentration of the tracer molecule D77 (600 nM) and increasing concentrations of unlabeled ligands **(A)** rimonabant, **(B)** HU-210, **(C)** CP 55,940, **(D)** 2-AG, **(E)** anandamide (AEA), **(F)** HU-308, and **(G)** SR 144528. Data were globally fitted to the competition association model using GraphPad Prism 9.2 to simultaneously calculate *k*_on_ and *k*_off_ values of the unlabeled competitors. Graphs show competition association curves from a single experiment representative of ≥3 experiments conducted independently.

**FIGURE 8:**
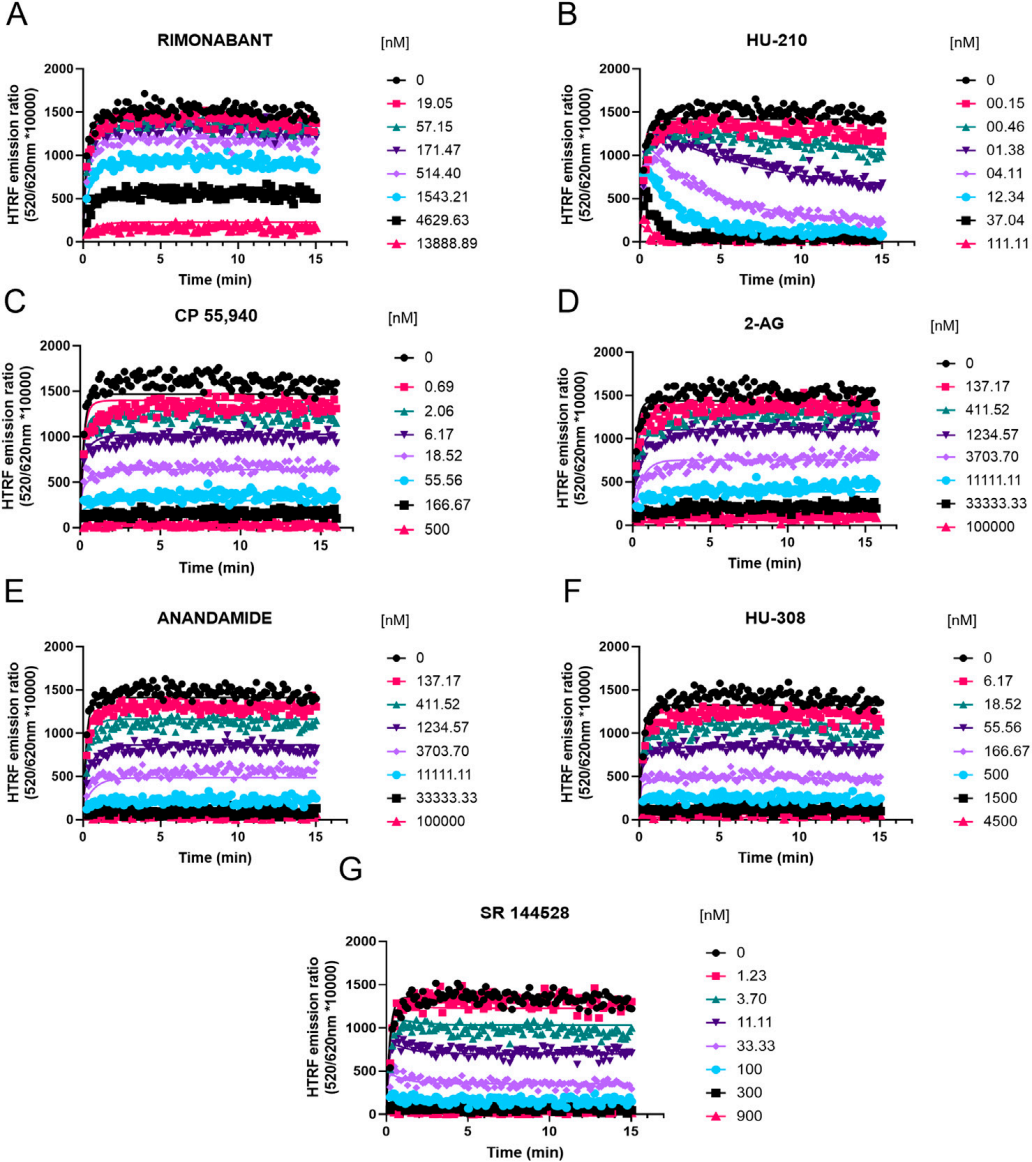
Competition kinetic binding curves of cannabinoid ligands at 37°C competing with D77 for the CB2R. Experiments were conducted using a fixed concentration of the tracer molecule D77 (900 nM) and increasing concentrations of unlabeled ligands **(A)** rimonabant, **(B)** HU-210, **(C)** CP 55,940, **(D)** 2-AG, **(E)** anandamide (AEA), **(F)** HU-308, and **(G)** SR 144528. Data were globally fitted to the competition association model using GraphPad Prism 9.2 to simultaneously calculate *k*_on_ and *k*_off_ values of the unlabeled competitors. Graphs show representative curves from four experiments conducted independently.

Having characterized the kinetics of D77 binding to CB1R and CB2R, we then evaluated its use as a tracer for the determination of the kinetics of unlabeled cannabinoid receptor ligands. The competition between the unlabeled compounds and D77 resulted in a concentration-dependent inhibition of D77 tracer binding in both the case of CB1R (see Figure 7) and CB2R (see Figure 8). The HU-210 competition curves exhibit the characteristic overshoot phenomenon, observed when the tracer first binds the receptor and then is displaced by the competitor. This reveals the slow dissociation profile of the competitor compound, HU-210, relative to the tracer. This effect was more prominent in the case of CB2R due an apostrophe to HU-210’s much slower rate of dissociation at this receptor subtype, relative to the tracer (see Figures 7B, 8B). The majority of the competition association curves show a gradual increase in D77 binding, indicating faster dissociation of the competing compounds, apart from SR 144528 binding the CB2R. As shown in Tables 3, 4, we report the kinetic association and dissociation rate constants (*k*_on_ and *k*_off_) and residence times (Rt = 1/*k*_off_) of the unlabeled agonists and antagonists binding to both CB1R and CB2R.

**TABLE 3:** Kinetic parameters calculated from the Motulsky and Mahan experimental approach for the cannabinoid compounds tested at CB1R at 37°C. The data shown are mean ± SEM from four experiments conducted independently (except for 2-AG, where N = 3).

CB1R (37°C)	*k*_on_ (M^−1^ min^−1^)	*k*_off_ (min^−1^)	Residence time (Rt)		p*K*_d_
			(min)	(s)	
Rimonabant	(5.03 ± 0.54) × 10^8^	2.23 ± 0.14	0.45 ± 0.03	27 ± 2	8.35 ± 0.06
HU-210	(3.79 ± 0.10) × 10^8^	0.85 ± 0.02	1.18 ± 0.03	71 ± 2	8.65 ± 0.01
CP 55,940	(1.54 ± 0.10) × 10^8^	5.63 ± 0.34	0.18 ± 0.01	11 ± 1	7.44 ± 0.03
2-AG	(1.40 ± 0.47) × 10^6^	6.74 ± 0.44	0.15 ± 0.01	9.0 ± 0.5	5.27 ± 0.15
Anandamide	(2.39 ± 0.46) ×10^6^	5.37 ± 0.63	0.19 ± 0.02	12 ± 1	5.64 ± 0.03
HU-308	(6.61 ± 0.81) × 10^5^	2.98 ± 0.35	0.35 ± 0.04	21 ± 2	5.35 ± 0.01
SR 144528	(1.35 ± 0.24) × 10^7^	4.99 ± 0.73	0.21 ± 0.03	13 ± 2	6.43 ± 0.01

**TABLE 4:** Kinetic parameters calculated from the Motulsky and Mahan experimental approach for the cannabinoid compounds tested for CB2R at 37°C. Data are expressed as the mean ± SEM of four experiments conducted independently.

CB2R (37°C)	*k*_on_ (M^−1^ min^−1^)	*k*_off_ (min^−1^)	Residence time (Rt)		p*K*_d_
			(min)	(s)	
Rimonabant	(9.20 ± 1.25) × 10^6^	6.37 ± 0.75	0.16 ± 0.02	10 ± 1	6.15 ± 0.03
HU-210	(2.62 ± 0.24) × 10^8^	(5.1 ± 0.29) × 10^−2^	19.84 ± 1.13	1,190 ± 67	9.71 ± 0.05
CP 55,940	(4.38 ± 0.48) × 10^8^	1.85 ± 0.14	0.55 ± 0.04	33 ± 3	8.37 ± 0.02
2-AG	(1.21 ± 0.18) × 10^7^	(1.44 ± 0.18) × 10^1^	0.07 ± 0.01	4.4 ± 0.7	5.92 ± 0.04
Anandamide	(9.69 ± 0.37) × 10^6^	6.15 ± 0.66	0.17 ± 0.02	10 ± 1	6.20 ± 0.04
HU-308	(5.58 ± 0.34) × 10^7^	1.53 ± 0.09	0.66 ± 0.04	40 ± 2	7.56 ± 0.02
SR 144528	(2.47 ± 0.16) × 10^8^	0.69 ± 0.03	1.46 ± 0.07	87 ± 4	8.55 ± 0.03

For CB1R, the fastest associating compounds were rimonabant, HU-210, and CP 55,940, displaying *k*_on_ values of 5, 3.8, and 1.5 × 10^8^ M^−1^ min^−1^, respectively, followed by SR 144528 (*k*_on_ = 1.4 × 10^7^ M^−1^ min^−1^). The two endocannabinoid compounds 2-AG and AEA showed similar slower association rate constants (*k*_on_ = 1.4 and 2.4 × 10^6^ M^−1^ min^−1^, respectively). The compound with slowest association was a CB2R selective binder, HU-308, with a *k*_on_ value of 6.6 × 10^5^ M^−1^ min^−1^, which is consistent with its lower affinity for CB1R.

Regarding the dissociation profile of the compounds at CB1R, the fastest dissociating compounds were 2-AG, CP 55,940, AEA, and SR 144528, therefore displaying the shortest residence times at this receptor subtype of ∼10 s. HU-210 exhibited the longest residence time (slowest *k*_off_) of 71 s, followed by the inverse agonist rimonabant (Rt = 27 s) and HU-308 (Rt = 21 s).

The competition binding approach has revealed a remarkably small difference between the kinetic dissociation parameters of these ligands, with only the agonist HU-210 displaying a relatively slow off-rate from the CB1R compared to the rapidly dissociating tracer D77 and the endogenous agonists AEA and 2-AG.

For CB2R, the fastest associating compounds were CP 55,940, HU-210, and SR 144528, displaying *k*_on_ values of 4.4, 2.6, and 2.5 × 10^8^ M^−1^ min^−1^, respectively, followed by HU-308, which displayed a *k*_on_ value of 5.6 × 10^7^ M^−1^ min^−1^. The two endocannabinoid compounds 2-AG and AEA showed similar slower association rate constants (*k*_on_ 1.2 × 10^7^ and 9.7 × 10^6^ M^−1^ min^−1,^ respectively), similar to rimonabant (*k*_on_ of 9.2 × 10^6^ M^−1^ min^−1^).

Regarding the dissociation profile of the compounds, those with fastest dissociation were 2-AG, rimonabant, and AEA, therefore displaying the shortest CB2R residence times of ∼4 s (2-AG) and ∼10 s (both rimonabant and AEA). CP 55,940, HU-308, and SR 144528 exhibited slower dissociation rates, with residence times of 33 s, 40 s, and 87 s, respectively. By far, the compound with slowest dissociation was HU-210, with a residence time of 20 min.

Competitive association binding experiments using the D77 tracer were performed at 25°C, and the resulting *k*_on_, *k*_off_, and *pK*_d_ values for both CB1R and CB2R are provided in Supplementary Tables S4, S5. As expected, the association and dissociation rates at 25°C were slower than those observed at 37°C (Tables 3, 4). Of note, the temperature effect varied across the tested compounds, leading to a different rank order for some of them. For example, at CB1R, rimonabant exhibited the longer residence time at 25°C (171 s), followed by HU-210 (160 s), whereas at 37°C, HU-210 was the compound displaying the longest residence time (71 s), followed by rimonabant (27 s).

### Kinetic parameters correlate differently for CB1R and CB2R

**FIGURE 9:**
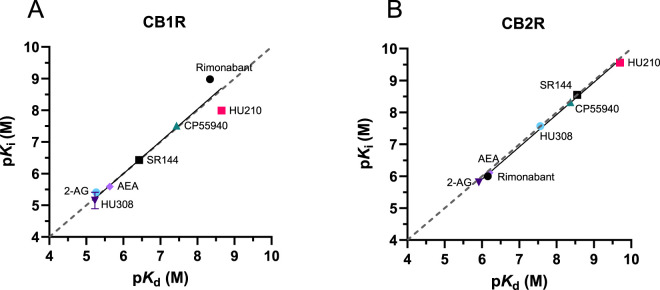
Kinetic versus equilibrium affinity estimates. Correlation between affinity values obtained from equilibrium displacement (p*K*_i_) and kinetic binding experiments (p*K*_d_) for the seven test compounds at **(A)** CB1R and **(B)** CB2R. p*K*_i_ values were taken from D77 competition binding experiments at equilibrium (see Figure 6). The values comprising the kinetically derived *K*_d_ (*k*_off_/*k*_on_) values were taken from the experiments shown in Figures 7, 8. The gray dashed line indicates the line of identity (y = x), representing perfect agreement between p*K*_i_ and p*K*_d_ values.

The equilibrium dissociation constants for the tested cannabinoid compounds were calculated from the kinetic association and dissociation rates from kinetic experiments (kinetic *K*_d_; *K*_d_ = *k*_off_/*k*_on_) and from the equilibrium displacement data (*K*_i_). Both values were compared for the binding of the compounds to both CB1R and CB2R. As shown in Figure 9, the kinetic *K*_d_ affinity values generated showed a strong correlation with the *K*_i_ values obtained from the equilibrium displacement binding; Pearson correlation coefficients (r) of 0.97 (P = 0.0004) and 0.99 (P < 0.0001) were obtained at CB1R and CB2R, respectively. These results show that the association and dissociation rates obtained from the association competition experiments are consistent with the affinity values obtained from equilibrium competition data, validating our approach using the tracer D77.

We performed a correlation analysis using the kinetic parameters (*k*_on_ and *k*_off_) versus affinity values obtained for our test set of compounds, in order to explore the role of association and dissociation constant rates in dictating the affinity of the ligands for CB1R and CB2R.

**FIGURE 10:**
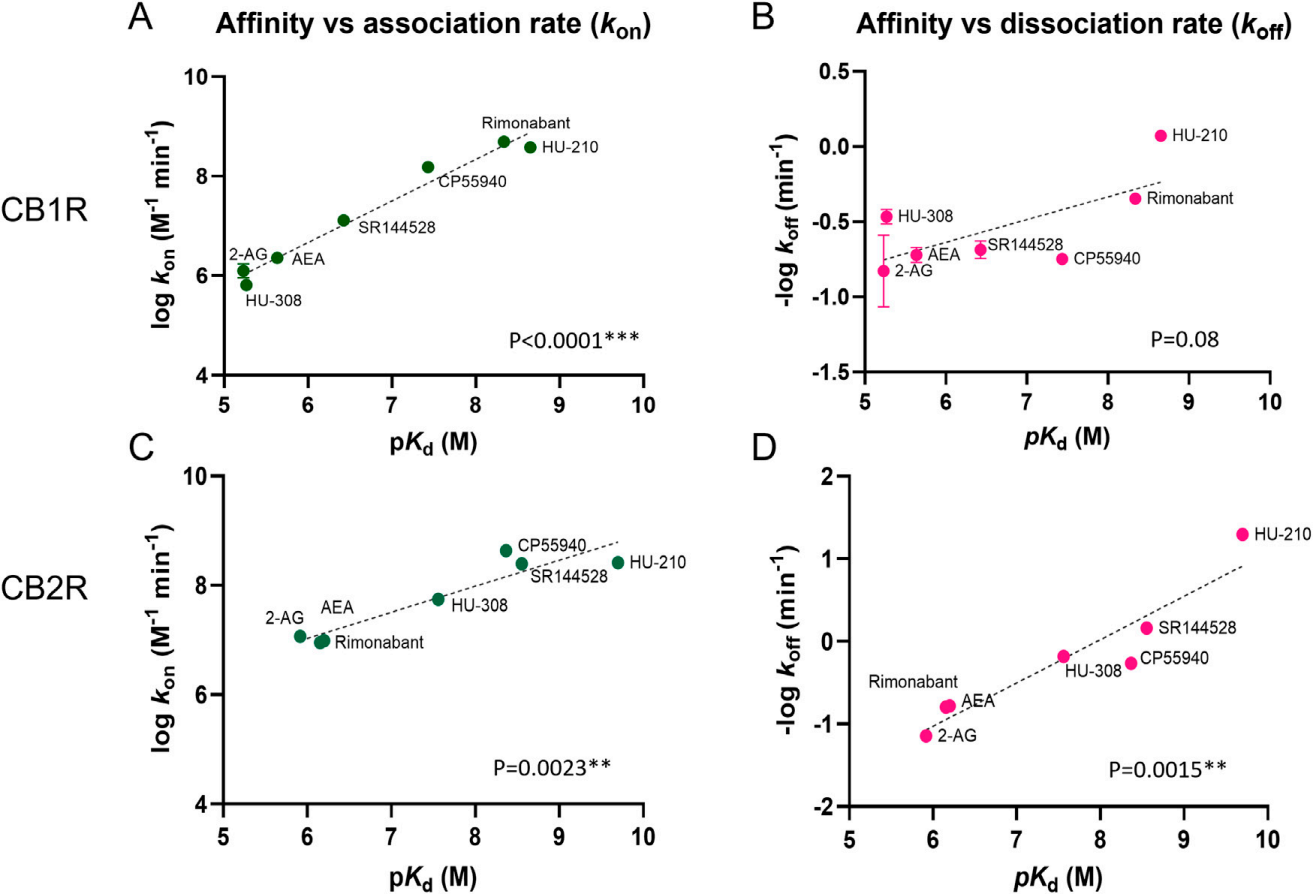
Correlation plots of equilibrium and kinetic parameters of cannabinoid compounds at CB1R and CB2R. Correlation between negative logarithmic transformation of affinities (−log *K*_d_) and logarithmic **(A)** association rate (log *k*_on_) and **(B)** dissociation rate (log *k*_off_) for CB1R ligands. Correlation between negative logarithmic transformation of affinities (−log *K*_d_) and logarithmic **(C)** association rates (log *k*_on_) and **(D)** dissociation rates (log *k*_off_) for CB2R ligands. Correlation analysis was carried out using a Pearson correlation analysis (two-tailed). Data shown are the mean and SEM of four independent experiments.

The affinities and association rates of compounds show a strong correlation at both CB1R and CB2R when performing a correlation analysis of their logarithmic transformations (p*K*_d_ vs. log *k*_on_), whereas the dissociation rates, expressed as negative logarithmic transformations, are only significantly correlated with ligand affinity values for CB2R (see Figure 10). These results suggest that *k*_on_, the association rate constant rather than *k*_off_, the dissociation rate constant, is the biggest determinant of receptor affinity for CB1R, whereas both *k*_on_ and *k*_off_ parameters dictate the affinity for CB2R binding. The correlation between the affinity and dissociation rates for the CB2R, found with the selected compounds in this study, contrasts with the results published previously with a different set of CB2R binding compounds (Martella et al., 2017), where no significant correlation was reported between affinity and *k*_off_ values.

## Discussion

In this study, we aimed to develop a fluorescent ligand binding assay for profiling the kinetic parameters of compounds binding to the orthosteric binding sites of CB1R and CB2R. The motivation for this was to advance on the existing radioligand binding techniques currently available for this purpose.

### Development of TR-FRET ligand-binding assay for cannabinoid receptors

Previously, we reported a TR-FRET ligand binding assay for CB2R (Sarott et al., 2020; Gazzi et al., 2022). In this study, we adapted the assay format for CB1R using genetic engineering to introduce an SNAP-tag to incorporate the donor, terbium cryptate. This ensured that our assay reported binding to only the overexpressed receptor subtype, avoiding tracer specificity issues seen in the radioligand binding assay. TR-FRET is a well-established approach, but it is often underappreciated as it only reports a signal based on proximity, providing ultimate assay specificity irrespective of the selectivity of the tracer employed. This improves the specific/non-specific signal ratio compared to traditional radioligand binding assays, where all ligands remaining after filtration contributes to the total signal, including the ligand which remains bound to unspecific sites of the cell membrane, or the filters themselves.

However, for CB1R, the truncation of the N-terminus was necessary to shorten the donor–acceptor distance and facilitate the FRET, a process we recently successfully employed to profile high-affinity fluorescent probes binding to CB1R (Mach et al., 2024). Although there is always a consideration that any receptor modification may affect its biogenesis, ligand binding, or signaling properties, our imaging, binding, and functional data strongly suggest that the distal N-terminal residues are not in any way impacting the trafficking or binding/activation capability of the receptor (see Supplementary Figures S2-S4). This same truncation strategy could be applied to develop fluorescent ligand binding assays for other receptors that have an exceptionally long N-terminus.

During our research, another group published TR-FRET equilibrium assays using full-length SNAP-tagged CB1R in combination with a proprietary red fluorescent ligand called CELT-335, which was also used for CB2R (Raïch et al., 2021). In comparison, our own fluorescent ligands did not yield a TR-FRET binding signal at the full-length SNAP-CB1R. The reason for this discrepancy is most likely due to differences in the fluorescent tracer used, both in terms of the linker (and hence how the conjugated fluorescent acceptor moiety may be positioned relative to the terbium donor on the SNAP tag) and the spectral properties of the fluorophore. CELT-335 is presumably a relatively bright fluorophore with the excitation/emission peaks of 646 nm and 662 nm, respectively (Navarro et al., 2023), whereas NBD (D77’s fluorophore) has excitation/emission peaks at 467 nm and 538 nm, respectively, and is relatively “dim.” The Förster radius (R_0_; the distance at which FRET efficiency is 50%) between a given donor and acceptor pairing is heavily influenced by the length of the linker and the properties of the acceptor fluorophore (Chen et al., 2006; Braslavsky et al., 2008), such as its molar extinction coefficient (ε) and the overlap between donor emission and acceptor absorbance (Chen et al., 2006; Braslavsky et al., 2008). These factors could account for a difference in R_0_ between the terbium cryptate donor and either the D77 or CELT-335 fluorophores, which would allow FRET for the full-length CB1R:CELT335 pairing but not the full-length CB1R:D77 pairing.

### D77 is an excellent fluorescent tracer for equilibrium ligand binding to CB1 and CB2

Our secondary aim was to develop a kinetically fast fluorescent ligand that would serve as an ideal tracer to profile other compounds binding to CB1R and CB2R. In order to achieve this aim, we synthesized and developed D77, a fluorescent derivative of Δ^8^-THC, as an optimal fluorescent tracer for cannabinoid receptors. D77 performed extremely well in terms of its ability to accurately measure the affinity of the non-fluorescent competitor compounds. Given its ease of use and our ability to scale up the testing throughput, through use of a commonly available plate reader with injectors and TR-FRET capability, this assay employing D77 effectively replaces radioligand binding assays previously employed to screen for ligands binding to cannabinoid receptors.

### Fluorescent tracer D77 applicability to kinetic studies

Crucially, D77, based on Δ^8^-THC, has a balanced affinity for both CBR subtypes, and it exhibits a more rapid *k*_off_ over the previously described CB2R tracers (Sarott et al., 2020; Gazzi et al., 2022; Kosar et al., 2023; Kosar et al., 2024). This improves the assay performance and allows more accurate and precise estimates of the kinetic parameters of more rapidly dissociating compounds (Georgi et al., 2019; Sykes et al., 2019).

The kinetic parameters for a number of compounds obtained with D77 are similar to those obtained earlier at CB2R using a radioligand kinetic binding assay at 25°C (Martella et al., 2017), where they reported similar residence times (CP 55,940: 5 min; HU-308: 4.2 min; SR 144,528: 8.7 min; AEA: 1.4 min; and 2-AG: 0.31 min) to those in our study (CP 55,940: 3.2 min; HU-308: 2.7 min; SR 144,528: 6.3 min; AEA: 0.8 min; and 2-AG: 0.5 min). Of note, D77 served our interest of developing an assay capable of determining the kinetic parameters of unlabeled ligands at physiological temperature. Moreover, the use of injectors overcomes the challenge of working at 37°C, which necessitates the collection of early time points, due to the fast association and dissociation rates observed for both the tracer D77 and the tested ligands.

### Measuring kinetic parameters of cannabinoid ligands at physiological temperature creates opportunities for improving compound efficacy under conditions of limited diffusion

**FIGURE 11:**
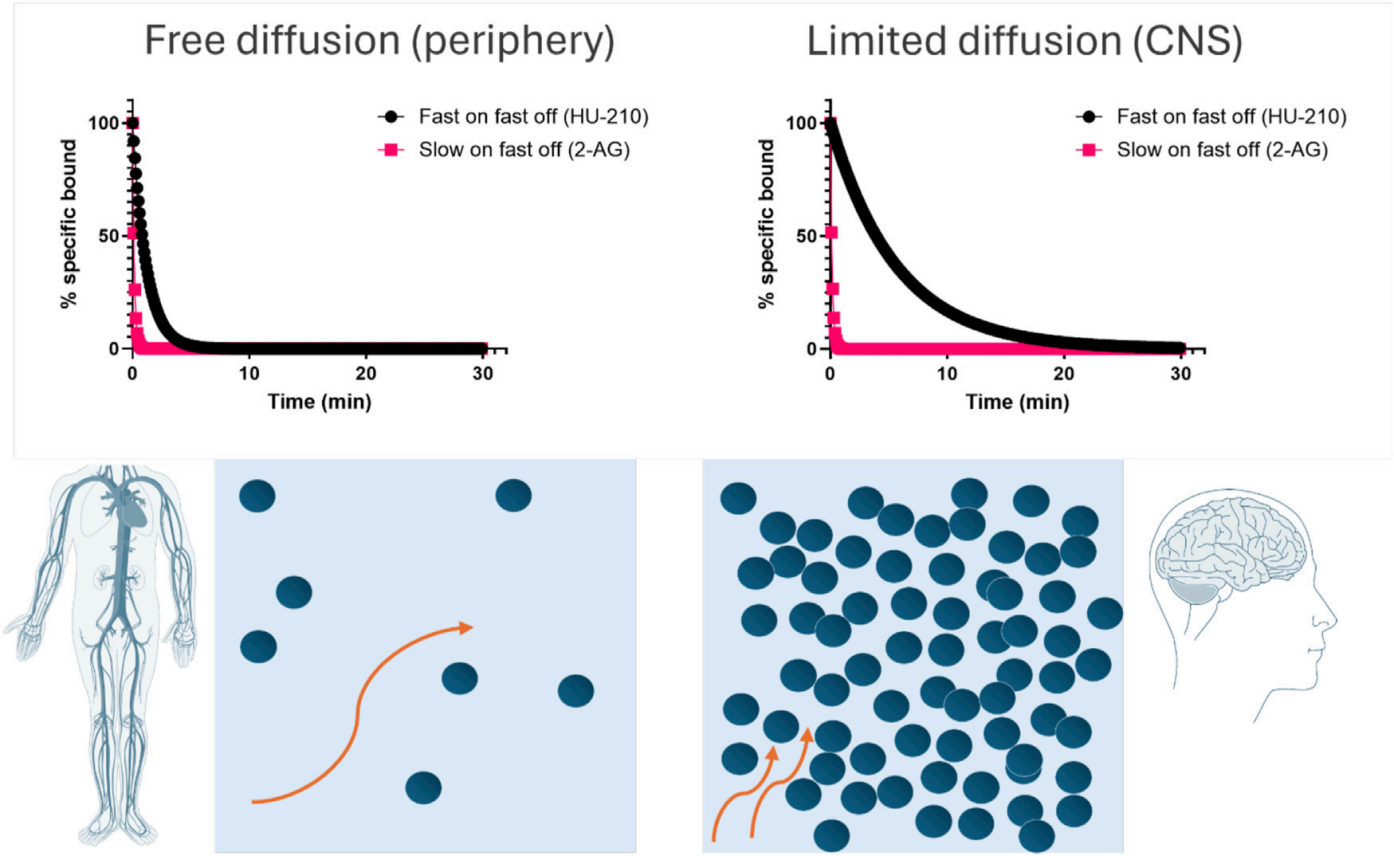
Consequences of rebinding on the apparent reversal of CB1R occupancy in the brain and periphery. Simulated target reversal rates were derived under conditions of limited diffusion based on the association (*k*_on_) and dissociation (*k*_off_) rates determined in competition kinetic binding experiments. All kinetic parameters used for these plots are taken from Table 3. For simulation purposes, the reversal rate *k*_r_ was based on the model of an immunological synapse (Coombs and Goldstein, 2004). *k*_r_ values are calculated using the following equation *k*_r_ = *k*_*off*_/(1+ *k*_*on*_ *R/k-), where *k*_off_ is the dissociation rate from the receptor, *k*_on_ is the association rate onto the receptor, [R] is the surface receptor density fixed at 1 × 10^11^ cm^−2^ for CB1R calculations, and k- is the diffusion rate of the synaptic compartment into bulk aqueous, fixed at 1.2 × 10^−5^ cm/s.

We successfully determined the kinetic binding parameters of seven different reference compounds binding to CB1R and CB2R. The kinetic parameters determined at 25°C (see Supplementary Tables S4, S5) and 37°C (see Tables 3, 4) reveal important differences in the residence time of the compounds tested at CB1R and CB2R, where, for example, HU-210 displayed a much longer residence time at CB2R (over 30 min longer). These findings strongly support the development of new approaches that enable kinetic characterization of ligand–receptor binding at physiological temperatures, allowing more accurate and detailed preclinical prediction models of drug action to be formulated. For example, if a particular receptor is mainly expressed in the brain, the prediction of drug binding in this compartment over time, through implementation of a rebinding model, will be more useful to identify optimal drug dosing (Sykes et al., 2017). In view of HU-210’s rapid association and slow off rate, it is intriguing that it has been shown to exhibit a noticeably longer duration of action in preclinical animal studies (Hruba and McMahon, 2014) and thus may possess unique beneficial qualities when it comes to behavioral and neurobiological alterations, compared to THC and other cannabinoids (Farinha-Ferreira et al., 2022). In contrast, the endogenous cannabinoids exhibit slow association and a much more rapid dissociation from both receptor subtypes. The potential for rebinding to affect the pharmacodynamic properties of ligands binding to CB1R in the brain and periphery has been documented in previous publications (Vauquelin, 2016; Terry et al., 2009), and its effect on apparent receptor reversal of the endogenous agonist 2-AG and the synthetic agonist HU-210 is illustrated in Figure 11. Under conditions of high receptor density and limited diffusion, such as those found in the synaptic environment of the brain, we can expect HU-210, which possesses a relatively rapid association rate, to occupy receptors for much longer than in the periphery, where diffusion occurs more freely. In contrast, under identical conditions, the endogenous ligand 2-AG, which has a much slower association rate and more rapid dissociation rate, would be expected to reverse more rapidly with very little influence of receptor density or diffusion on this process.

### Limitations of the study

Previous TR-FRET binding assays have been successfully developed to profile the kinetics of ligands binding GPCRs (Schiele et al., 2015; Sykes et al., 2017). One limitation of this new CB1R assay format is that the receptor needs to be truncated to incorporate the SNAP-tag at the N-terminus close to the orthosteric binding site. Indeed, fluorescent ligand binding to human CB1R has been described previously using TR-FRET and an SNAP-tagged full-length CB1R (Raïch et al., 2021); however, it is unclear if kinetic assays with the reported tracer would be feasible, as their binding kinetic parameters were not reported. Unfortunately, none of the fluorescent ligands reported in this study showed detectable signals with the full-length CB1R. Although there may be some tertiary structure present in the first 90 residues of the N-terminus, the absence of this region did not influence the pharmacology of the receptor. The second limitation is the necessity of an SNAP-tagged receptor. This effectively prevents the use of this ligand binding assay with endogenously expressed untagged receptors. Although a genetically engineered mouse model with an SNAP-tagged GLP-1 receptor has been reported (Ast et al., 2023), such models are not available for CB1R or CB2R.

## Conclusion

The novel TR-FRET-based method we outline utilizing the probe D77 (a fluorescent derivate of Δ^8^-THC) constitutes a simple and superior alternative to radioligand binding methodologies to determine the equilibrium and kinetic binding of compounds for cannabinoid receptors at physiological temperature. Investigating the kinetic parameters of prospective cannabinoid drug candidates could help us identify essential factors for refining their design and lead to the discovery of more effective medicines to target these receptors.

## Data Availability

The original contributions presented in the study are included in the article/Supplementary Material; further inquiries can be directed to the corresponding authors.
